# Maintaining Optimal Mammary Gland Health and Prevention of Mastitis

**DOI:** 10.3389/fvets.2021.607311

**Published:** 2021-02-17

**Authors:** František Zigo, Milan Vasil', Silvia Ondrašovičová, Jana Výrostková, Jolanta Bujok, Ewa Pecka-Kielb

**Affiliations:** ^1^Department of Nutrition and Animal Husbandry, University of Veterinary Medicine and Pharmacy, Košice, Slovakia; ^2^Department of Biology and Physiology, University of Veterinary Medicine and Pharmacy, Košice, Slovakia; ^3^Department of Food Hygiene, Technology and Safety, University of Veterinary Medicine and Pharmacy, Košice, Slovakia; ^4^Department of Animal Physiology and Biostructure, Wrocław University of Environmental and Life Sciences, Wroclaw, Poland

**Keywords:** dairy cows, mastitis, somatic cells count, nutrition, bedding, dry period, treatment

## Abstract

In dairy industry, quality of produced milk must be more important than quantity without a high somatic cells count (SCC) or pathogens causing mastitis of dairy cows and consumer diseases. Preserving the good health of dairy cows is a daily challenge for all involved in primary milk production. Despite the increasing level of technological support and veterinary measures, inflammation of the mammary gland–mastitis, is still one of the main health problems and reasons for economic losses faced by cow farmers. The mammary gland of high-yielding dairy cows requires making the right decisions and enforcing the proper measures aimed at minimizing external and internal factors that increase the risk of intramammary infection. Due to the polyfactorial nature of mastitis related to its reduction, the effectiveness of commonly used antimastitis methods tends to be limited and therefore it is necessary to find the areas of risk in udder health programs and monitoring systems. Only by implementing of complete udder health programs should be accompanied by research efforts to further development these complete udder health control. The present review analyses the current knowledge dealing with damping and prevention of mastitis include SCC control, proper nutrition, housing and management, milking and drying as practiced in dairy farming conditions. This information may help to improve the health of the mammary gland and the welfare of the dairy cows as well as the production of safe milk for consumers.

## Introduction

Ruminant milk is a traditional raw material for the production of a range of dairy products that are unique in their composition. Many of them can be classified as functional foods in different geographical and social localities. However, European Union regulations oblige producers to obtain milk only form healthy animals in order to increase consumers safety, what however may limit milk production and consumption (1–4).

A number of factors influence the health status of ruminants in large dairy herds. Both single factors and their combination create the conditions in which the virulence of pathogens, especially bacteria, break the host's immunity. Various organ diseases may be induced among others by inadequate housing hygiene, poor nutrition, and mistreatment and when many of the animals are affected they may be defined as so-called production diseases (5).

In publications on dairy farming, mastitis, laminitis, and metritis are distinguished as the main three production diseases. The US Animal Welfare Council concluded that production diseases are currently considered the most serious problem in dairy farming, causing, in addition to deterioration of dairy animals health and welfare, huge economic losses (6).

Despite the increasing quality of zootechnical control and better hygiene of milk production, mastitis remains the most serious and demanding disease of dairy cows with significant negative economic impact. The negative economic consequences of clinical or subclinical mastitis include a decrease in milk production and lower price for milk with high SCC, increased rate of culling, and higher cost of veterinary treatment, which can climb up to 185 EUR/cow (7). A survey conducted by Turk et al. (8) showed that 23% of cows leaving the herds too early are culled because of udder health problems.

Equally important negative impacts are related to the poor technological quality of raw milk used in the dairy industry, and the presence of mastitis pathogens and their toxins in milk and dairy products (9, 10). Based on over 70 years of systematic studies on mastitis in ruminants, a general thesis is accepted that the disease is polyethiological and multifactorial, and therefore it requires a comprehensive approach to reduce its incidence (11–13).

Methods of disease prevention and control must be based primarily on the results of targeted diagnostics, including history data, to reveal the clinical status of the udder, and confirm the extent of anatomical and pathophysiological changes in the mammary gland (MG) (14, 15).

An overview analyzes antimastitis measures aimed at damping and prevention of mastitis include SCC control, proper nutrition, housing and management, milking and drying as practiced in dairy farming conditions to improve the health and welfare of the cows.

### Causes of Mastitis

According to Holko et al. (16), the causes of mastitis can be fundamentally divided into two groups. In the first group, inflammations of the MG and milk ducts are caused by microbial. In the second group are applied incorrect technological procedures during milking, metabolic disorders, udder injuries and various stress factors in the development of mastitis.

**Figure 1:**
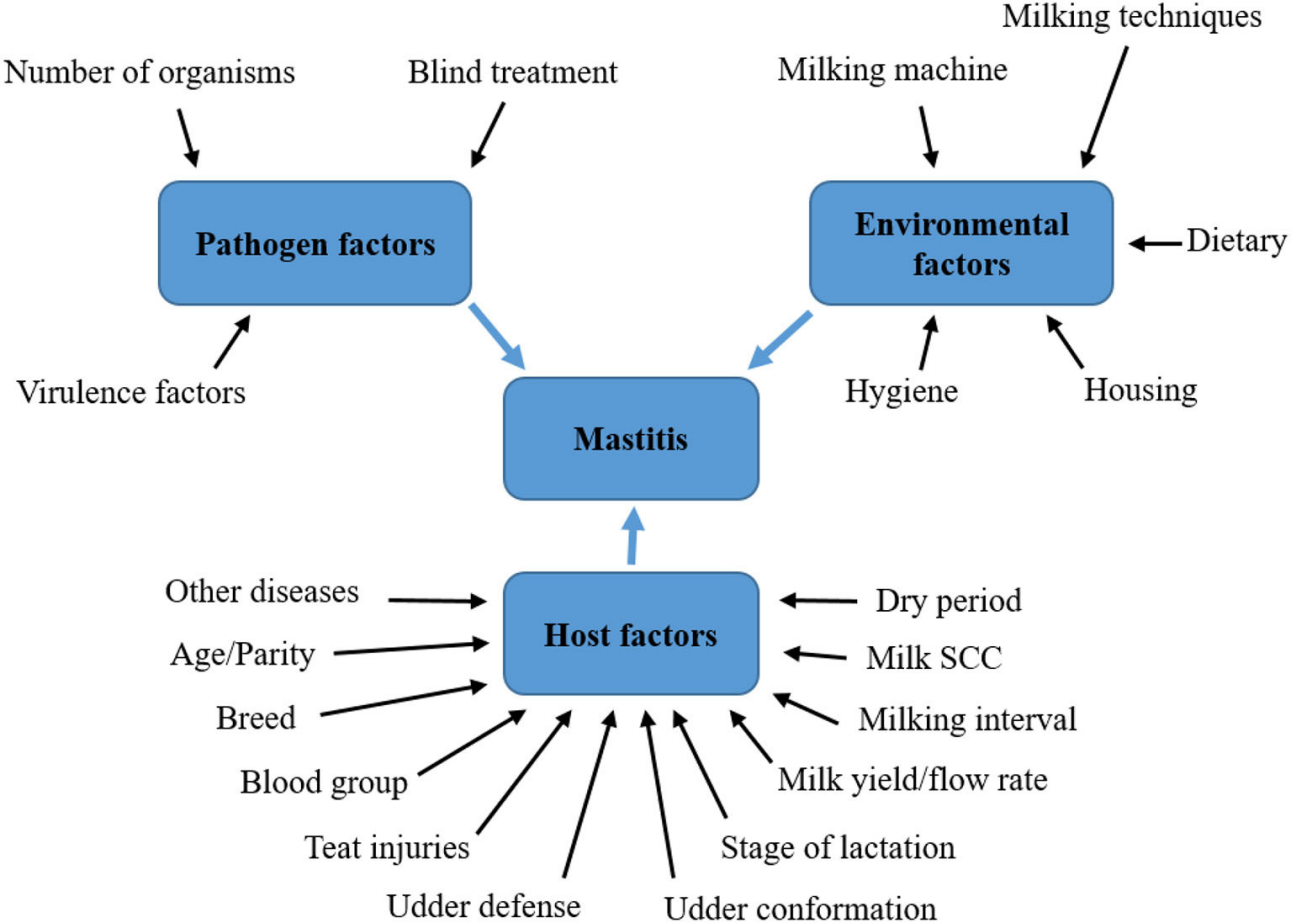
Factors influencing the development of mastitis in dairy cows. Soure: Adapted from Abebe et al. (17).

Acceptance of the mutual relationship between the infectious agent and the dairy cow organism is of fundamental importance in influencing all factors of the external environment, while the susceptibility of the dairy cow to mastitis is also given by factors such as: age, order of lactation, its stage, milk yield, anatomical dispositions, but mainly by immunological condition and reactivity of the mammary gland. Due to the multitude of internal and external causes leading to mastitis, it should be considered a multifactorial disease [(17), Figure 1].

To date, over 137 different organisms have been identified as being causative agents of bovine mastitis, including bacteria, viruses, mycoplasma, yeasts and algae but bacteria remain the principle causative agents (95% of all IMI) of such complex (18, 19). Generally, every mastitis case is considered to be caused by one primary pathogen, because usually only one bacterial species is identified in milk samples from diseased glands. Nevertheless, simultaneous infections by two different pathogen species are not rare, and three pathogens may be found in a small proportion of cases.

The bacteria causing the most common forms of mastitis may be considered within two groups. Contagious pathogens (e.g., *Staphylococcus aureus, Streptococcus agalactiae* or *Streptococcus dysgalactiale*) (20). These organisms can survive and grow within the MG so that transmission of infection from infected to uninfected quarters and from cow to cow is most likely to occur during milking. Environmental pathogens thrive in the environment especially where cows' feces are involved. Of this group, *E. coli* is the most important with multiple strains of varying pathogenicity for animals and humans. Others include *Streptococcus uberis*, coagulase-negative staphylococci (CNS), *Corynebacterium* spp., *Pseudomonas* spp., *Serratia* spp., *Proteus* spp., *Pasteurella* spp., *Listeria* spp., *Leptospira* spp., *Yersinia* spp., *Enterobacter* spp., *Brucella* spp. and *Mycobacterium* spp. (21–25).

Pathogens can invade into MG in various ways. The most common way is penetration through teat orifice and teat duct as well as through microlesions and damaged skin of the udder (26, 27). With the exception of a few pathogens that can invade via the bloodstream (e.g., *Mycobacterium avium* ssp. *Paratuberculosis* or *Brucella abortus* from other organs. The entero-mammary pathway has been invoked to explain the transfer of gut lumen bacteria to the MG. It is speculated that bacteria taken up from the gut lumen by leucocytes such as dendritic cells or macrophages would be carried to the MG by phagocytes migrating to the MG by the haematogenous route, then making their way to the MG lumen to be finally shed in milk. It has been shown that translocation of bacteria from the gut lumen to milk in mononuclear leucocytes may occur in lactating mice for a short period after delivery (28).

The manifestations of the inflammatory process vary widely, as they depend on the degree of reaction of the udder tissue to injury or infection (29). The clinical manifestations of MG inflammation as well as its further course depend on the interplay between the innate resistance and adaptive immunity of the dairy cow and the type, concentration, and virulence of udder pathogens. If the MG has been infected with a large number of pathogens or more germs that are virulent and the host's defense systems have not been sufficient to control the infection, an clinical or chronic form of mastitis will develop (22, 30).

Clinical form of mastitis is characterized by a sudden onset, alterations in milk composition and appearance, decreased milk production, and the presence of the cardinal signs of inflammation in infected mammary quarters. In contrast, a lower concentration of pathogens with lower virulence leads to subclinical infections without visible symptoms in the udder or milk, but the milk production decreases and the SCC increases. According to Sharma et al. (21), subclinical mastitis is considered the most economically important type of mastitis because of its higher prevalence and long term devastating effects as compared to clinical mastitis. The symptoms of subclinical IMI may only be recognized from evidence of an elevated SCC. In some cows it may persist throughout lactation without presenting clinical signs, in others give rise to repeated episodes of (probably mild) clinical symptoms. In these circumstances, cows would be defined as cases of chronic mastitis. Whether subject to repeat clinical episodes or not, cow with chronic mastitis (especially with *S. aureus*) continue to shed organisms so present a risk of cross-infection at the time of milking.

In recent years, one of the most common microorganisms causing mastitis in dairy cows is *S. aureus* and coagulase negative staphylococci (CNS) (18). Bacteria *S. aureus* is a natural inhabitant of the skin of cows and humans, where it does no harm unless the cow s teat or the milker's and is cracked, when it can cause the wound to turn septic. If the organism is able to penetrate the teat in sufficient numbers the disease taken one of two clinical forms of IMI. Peracute staphylococcal mastitis can occur rarely, but especially in early lactation when the immune defenses of the cow are depressed. The cow becomes very ill with a high fever, depression, inappetence and may become comatose and die within 24 h of the onset of symptoms. The infected quarter is grossly swollen and extremely painful, which makes the cow very reluctant to move. The secretion from the infected quarter is usually a lood-stained, serous fluid. If the cow survives, blue gangrenous patches may appear on the quarter and proceed to blackened, oozing sores. Although the cow with peracute *S. aureus*. infection can be saved by an effective antibiotic, if caught in time, the quarter is almost invariably lost (20).

The more common form of *S. aureus* infection is less severe but chronic. The affected cow may not appear ill and the affected quarter may not be paiful. The foremilk may or may not show abnormalities (31). However, as with *Strep. agalactiae*, chronically infected quarters are sources of cross infection and become progressively less productive as scar tissue replaces secretory tissue. Treatment of *S. aureus* infection is complicated by the fact there are many strains and more and more of them are becoming resistant to more and more of the antibiotics within the veterinary armory (19, 32).

Coagulase-negative staphylococci are considered to be minor pathogens in dairy mastitis however, there is increasing work by authors to emphasize their role in the development of MG inflammation (23, 33–35). The increase of their occurrence in dairy farms occurs after the reduction of the occurrence of the main pathogens; the CNS that are present are characterized by increased resistance to commonly used antibiotics and disinfectants (34). Compared to *S. aureus*, CNS usually have a lower proportion of virulence factors but their essential factor of pathogenicity is the production of a biofilm and thus resist the applied disinfection and sanitation procedures. In addition, in their study, Nascimento et al. (36) confirmed that the CNS (*S. epidemidis, S. saprophyticus, S. hominis and S. aerletae*) which were isolated from cow mastitis, were resistant to the antibiotics used and were able to produce some of the staphylococcal enterotoxins. Haveri et al. (37) and Vasil et al. (38) consider the ability to produce staphylococcal enterotoxins to be an important virulence factor which is responsible for the development of, in particular, clinical forms of mastitis. Previous studies indicate that CNS with some virulence factors and multiple resistance, are very important in pathogenesis of mastitis in dairy cows.

### Immunocompetence in the Mammary Gland

**Figure 2:**
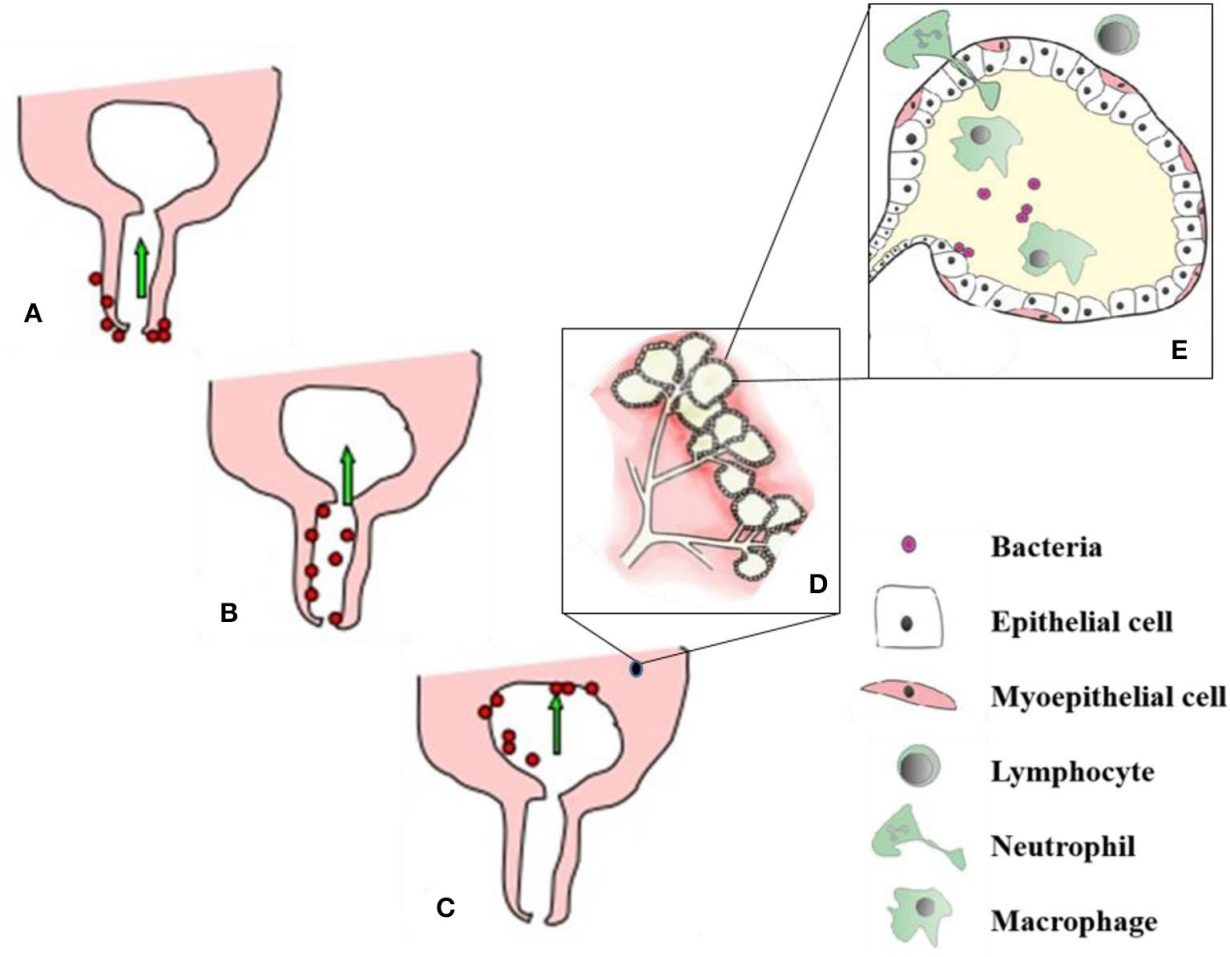
Process of udder infection. **(A)** – Organisms invade the udder through teat canal, **(B,C)** - Migrate up the teat canal and colonize all parts where the milk gets and finally setup the infection in mammary gland, **(D)** - alveoli and secretory mammary epithelial cells. After getting bacterial infection, cellular defense mechanism become active and phagocytic cells (from blood) effort to engulf and kill the bacteria, phagocytosis by products and release of bacterial toxins damage to the secretory mammary epithelial cells **(E)**. Soure: Adapted from Sharma and Jeong (21).

Immunocompetence in the mammary gland (MG) is a complex of non-immune anatomical factors, and a plethora of immune-mediated defense mechanisms that include innate and adaptive immune responses. Immunocompetence can vary during lactation, showing depression in the peripartum period due to the hormonal and metabolic stress of calving and milk production. Decreased immunity after calving with the negligence in an application of milking hygiene program, housing hygiene, nutrition and breeding work increases transmission of pathogenic microorganisms and the contamination of the mammary quarters (39). In most cases, mastitis-causing microorganisms enter the MG tissue and milk through the teat duct, from where they are transmitted and spread to remaining structures of the glandular tissue [(21), Figure 2].

The teat orifice and teat duct are therefore considered as one of the most important physical barriers of the defense system against the penetration of microorganisms into the mammary gland. The teat duct epithelium produces keratin that physically traps bacteria and blocks their migration to the mammary cistern. Keratin also has antimicrobial activity due to some bacteriostatic fatty acids (lauric, myristic, palmitoleic, and linoleic), as well as fibrous proteins that bind and damage the microorganism cell wall. Damage of keratin, perhaps as a result of incorrect intramammary therapy infusion or by faulty machine milking, has been reported to increase susceptibility of the teat canal to bacterial invasion and colonization (20, 40, 41).

One of the important barriers that affects the transfer of pathogens from the environment and may govern mastitis susceptibility is commensal microbiota residing in the udder. Bacteriocins produced by certain non-aureus Staphylococci and Corynebacterium species colonizing the teat apices and teat canals may inhibit growth of major mastitis pathogens. According to Bronzo et al. (39) commensal microbiota of MG can change immune responses through direct and indirect mechanisms, such as through bacterial secretion of antimicrobial compounds or through influencing the expression of genes hosts' immune responses.

Within complex ecosystems, ecosystem diversity can increase resiliency against an influx of external species by supporting favorable interactivity. The complexity of microbe to microbe communications concerning the functional properties of the mammary ecosystem are difficult to understand. It is essential to identify those bacterial species in the milk microbiota that contribute to mammary homeostasis and mastitis pathogen susceptibility (42).

The MG displays both innate and adaptive (or acquired) immune mechanisms that defend the tissue against pathogens. The innate immune system (IIS) is the first line of defense against pathogens after penetration through physical barrier of the teat canal and before the adaptive immune system comes into play, and it evolves into a highly effective host defense. Innate host-defenses depend on germline-encoded receptors that recognize conserved structures expressed by a wide range of microbes, and early induced cellular and soluble defenses (43).

A wide variety of innate immune components have been identified in colostrum and milk, including cellular defense components (e.g., leukocytes, neutrophils, macrophages) components contributing to humoral defense (e.g., complement system, immune-modulating factors, cytokines, lactoferrin, transferrin, lysozyme, and components of the lactoperoxidase/myeloperoxidase systems, oligosaccharides, gangliosides, reactive oxygen species, acute phase proteins), ribonucleases, and a wide range of antimicrobial peptides and proteins. These components of IIS respond quickly to microbes during early stages of infection and are tightly integrated with the adaptive immune system (44).

The adaptive immune system uses a diverse repertoire of antigen specific receptors expressed by clonally expanded B and T-lymphocytes to regulate or eliminate the signal elicited by recognition events. Additionally, the induced adaptive immune response has the capacity to establish antigen specific memory for a rapid and augmented response upon subsequent exposure to the same antigen (45).

Generally, the microorganisms located on the surface of the teat penetrate the udder during or after milking through the teat canal. Especially, after milking, the teat canal is open for 1–2 h or for the entire period between milking if damaged. This condition facilitates the entry of microorganisms from the environment, in particular from the dirty bedding. Microorganisms, after penetration of the MG, attack and colonize tissues. Some of the microorganisms spread to the higher parts of the glandular tissue of the MG when the cow moves after the milk has mixed in the milk cistern (22).

As mentioned, both innate and adaptive immune response are coordinating and operating together in very complicated pathways to provide the optimal defense against infections. After contacting the bacteria with leukocytes in the milk or the lining gland epithelium accompanied by exerting various virulence mechanisms and liberating toxins, irritation or even damage to MG epithelium and, thereby, activation of the IIS occur through the transcriptional activation of key response genes (41).

Inflammatory products from damaged epithelium induce locally located leukocytes and healthy MG epithelium to release several chemoattractants for the migration and recruitment of both bone marrow and circulating immune cells into the MG environment, mainly neutrophils. Proinflammatory cytokines are the main effectors to initiate the inflammatory responses at both local and systemic levels (46). They act in collaboration with transforming growth factors and several chemotactic factors to potently trigger circulation-into-MG migration of neutrophils via induction of vascular endothelial adhesion molecules expression. These processes lead to the recruitment of further leukocytes from blood, their passage to milk and an infiltration of udder tissue. The migration of immune cells during IMI plus desquamation of MG epithelia results in an increase of SCC accompanied with decreased milk production according to the severity of the process (42).

If the udder cannot be cleared from the invading microorganisms, persistently activated leukocytes may injure the intralobar ducts and the alveoli. Damage to the alveolar epithelial cells increases the permeability of the capillaries leading to further increase in the number of white blood cells in the infected tissues and to the influx of the minerals and clotting factors from the blood (14).

The interaction between the pathogenic microorganisms and the host's immune system leads to the coagulation and retention of milk, which results in the closure of the ducts. The activity of secretory cells is suppressed, the alveoli reduce in size, then secretory cells are destroyed and replaced by connective tissue (22, 29).

Trinidad et al. (26) using histological analysis of mammary tissue samples from primipary cows showed that percentages of alveolar epithelium and lumen in quarters infected with *Staph. aureus* were lower than those in uninfected quarters. Quarters infected with *Staph. aureus* also showed a greater percentage of interalveolar stroma than did uninfected quarters. Additionally, quarters infected with *Staph. aureus* exhibited significantly greater infiltration of leukocytes (mainly lymphocytes and neutrophils) compared with uninfected tissues.

### Damping and Control of Mastitis

A comprehensive approach is necessary to determine appropriate control and prevention measures in dairy farming for the production of high quality milk while maintaining udder health. It should be borne in mind that mastitis cannot be completely eliminated from the herd but only kept at the lowest possible incidence. Improving MG health at farm level is based on the application of two basic principles:

1. shortening the duration of an existing intramammary infection
2. reducing the incidence of new intramammary disease.

Since mastitis is a multifactorial disease, a successful breeder should have certain characteristics to apply these two basic principles; He must fully understand the complexities of the disease, know the principles of prevention and control, be motivated and determined, be able to motivate his employees and lastly, be able to put (comprehensive) knowledge into practice. Improving the health of the mammary gland and the production of quality milk can only be achieved through the application of broad-spectrum mastitis prevention and control programs (47).

Antimastitis measures must consider all aspects of both the external and the internal environments based on daily husbandry practices that affect the health of the dairy cow and its milk production. Required aspects include:

- the benefit of reducing the incidence of mastitis in the herd must be more than the cost of treating and controlling it,
- the choice of measures to control mastitis must be applicable throughout the herd,
- the measures in place must be effective against all mastitis pathogens (25, 48).

#### Somatic Cells Count Monitoring and Reduction

**Figure 3:**
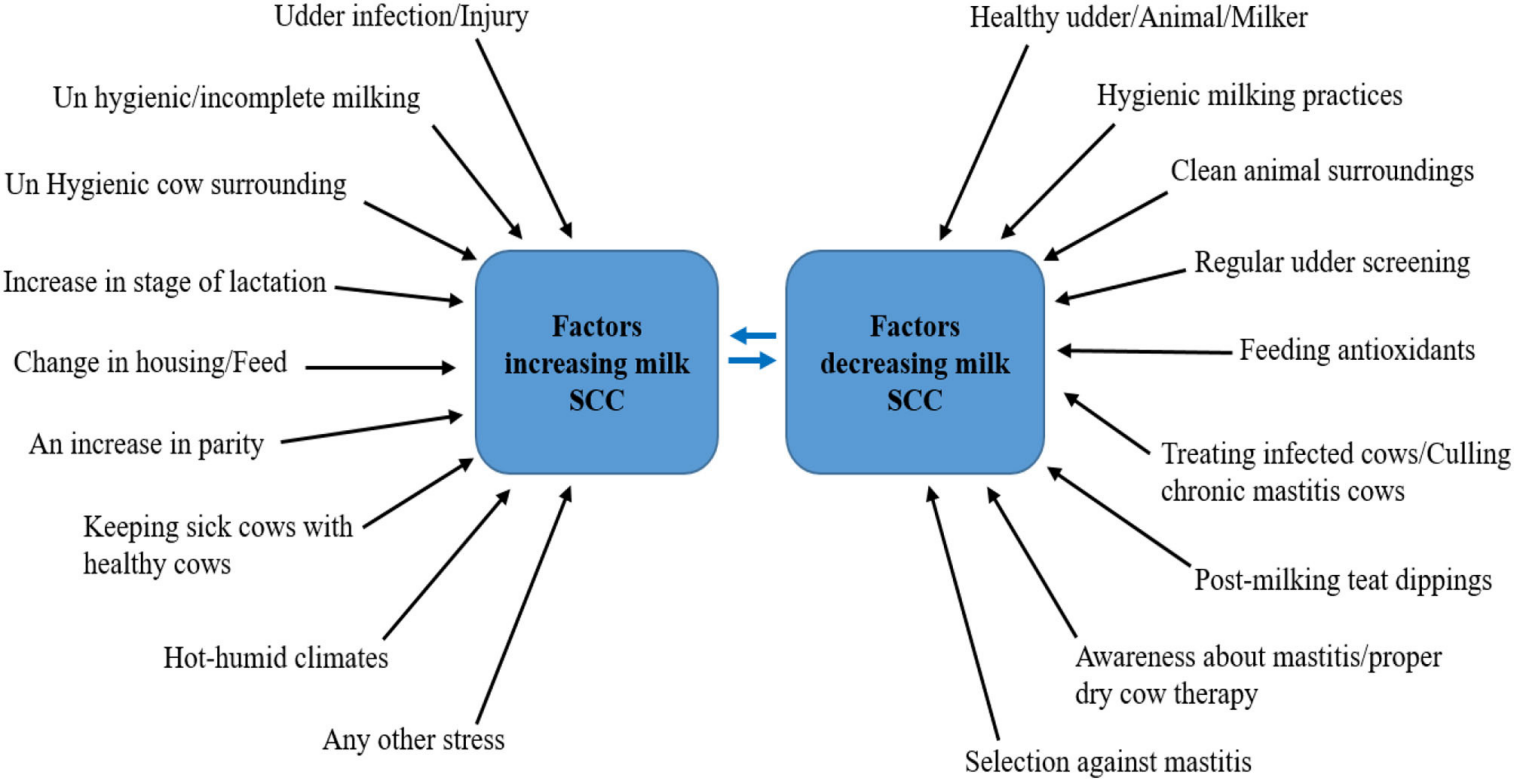
Factors and management practices affecting the release of milk SCC. Source: Adapted from Alhussien and Dang (49).

There are many factors and management practices that affect the release of milk SCC and can cause a decrease or increase in their levels [(28), Figure 3]. Researchers over the years have found associations between various management practices adopted on dairy farms and herd SCC (14, 15, 50, 51).

Low SCC milk production is an important tool for a good dairy economy. Somatic cells are predominantly white blood cells that are produced by the immune system to prevent inflammation of the mammary gland (mastitis). Inflammation of the mammary gland can have a clinical course (when changes in the udder and milk are visible), and subclinical as well when there are no visible symptoms, however, increased SCC in milk results to a decrease in milk production [(52), Table 1]. The increase in the number of cells in milk due to the inflammatory response can be enormous. From a base level of only 100.000 (10^5^) per mL, i.e., a cell count of 100, it may increase to as many as 100.000.000 (10^8^) per ml (a cell count of 100.000) in just a few hours, and many quarters rapidly reach a cell count of 10 billion (10^9^).

**Table 1:** Estimated milk losses due to increased SCC.

**SCC/ml**	**Loss of milk (%)**	**Losses of milk production per dairy cow/year (kg)**
100 000	3	180
200 000	6	360
300 000	7	450
400 000	8	550
500 000	9	590
600 000	10	635
700 000	10.5	680
800 000	11	725
900 000	11.5	750
1 000 000	12	770
1 600 000	12	770

*Source: Tongel and Mihina (52)*.

The system of mastitis control in a specific herd can be implemented through the control of SCC in a pool sample based on monthly reports from the performance control. If the pool somatic cells rise above 400 x 10^3^ in 1 mL within 2 months, then there is a problem with the classification of milk in the breeding, thus, requires solutions to the causes of the unfavorable condition (2).

The fastest way is to sort out cows with increased SCC. Such dairy cows should be milked last in special cans so that milk with elevated SCC (often contaminated with microorganisms) is not mixed with the remaining milk in the cooling tank. However, this is a short-term quick solution to reduce SCC in the pool sample. Often, breeders in the created group of dairy cows called “millionaires” (>1,000 x 10^3^ SCC), classify the chronically ill or incurable dairy cows in which no effect on SCC reduction or elimination of the mastitis-causing pathogen was observed even after multiple treatments. Additionally, dairy cows that repeatedly fail to respond to treatment are considered high-risk vectors for the transference of resistant strains to other dairy cows in the established group and the herd as well (21).

The breeder should consider their exclusion based on a good record and frequency of chronic and incurable mastitis caused by contagious pathogens in individual dairy cows in selected groups. The culling of dairy cows with recurrent mastitis and ineffective treatment represents a very effective way to reduce SCC as well as bacterial pressure in a herd, especially in contagious infections (53).

The second way is prevention, particularly the control of mastitis animals and their subsequent treatment. The distinction between new (mainly subacute and acute forms) and long-term inflammation is possible mainly based on daily diagnosis and evidence (54). Cows with severe infections will likely need veterinary intervention and require immediate and aggressive treatment with fluids, systemic and intramammary antibiotics, anti-inflammatories and calcium. But severe cases only occur 15% of the time; the other 85% are the mild and moderate cases where milk cultures are most informative (55).

**Figure 4:**
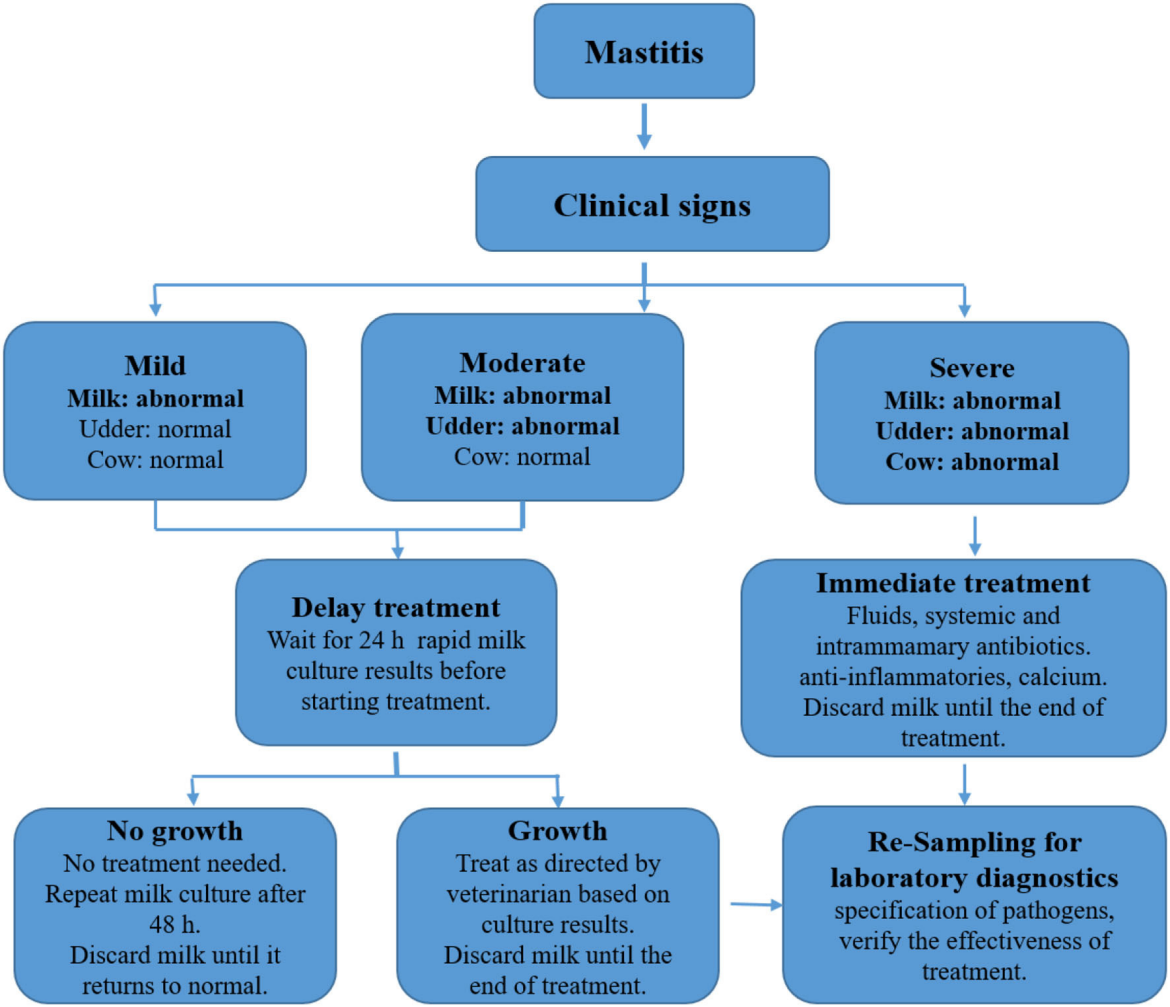
Determination of severity and treatment of mastitis according to clinical symptoms. Source: Adapted from Bargren (56).

In practical conditions, antimastitis protocols include sampling from suspected dairy cows for the purpose of rapid cultivation and differentiation of G– and G + bacteria in a thermobox directly on the farm. Zootechnics are in charge of the anamnesis and determination of the degree of severity of mastitis according to the clinical signs of inflammation (milk, udder, cow). After culturing the samples for 12 or 24 h, the result of the bacteriological examination is read and, based on the obtained result and the previous anamnesis, the attending veterinarian applies antibiotics [(56), Figure 4].

For the attending veterinarian, according to Tančin et al. (14) it is important to know if the cow has G^−^ and G^+^ bacteria in the udder. In the second case, antibiotic treatment is started after re-culturing the sample taken 24 h after the last application of antimicrobials. Based on the etiological agents, there are 1st, 2nd, and 3rd line antibiotics listed in the antimastitis protocols. The first-line antibiotics are novobiocin, penicillin, dihydro-streptomycin and neomycin. Second-line antimicrobial drugs are amoxycillin-based preparations. Third-line antibiotic is lincomycin-based preparation. When choosing intramammary formulations, it is necessary to start from the long-term monitoring of the pathogen sensitivity in a given herd, and individually evaluate the occurrence of new cases. The main task of the veterinarian in this scheme is to evaluate the effectiveness of individual drugs as well as to adjust the antimastitis protocol, e.g., in the case of some pathogens, to omit the first-line antibiotics, etc. The main advantages of this system are an increase in the effectiveness of antibiotic therapy and a reduction in the occurrence of resistant strains in breeding (55).

Only early diagnosis of mastitis with the cultivation of bacterial mastitis agents with the selection of a suitable antibiotic to which their highest sensitivity has been recorded increases the effect of the treatment with a positive impact on SCC reduction and restoration of the required milk production. This method is only long-term, but also more economical and efficient. Noteworthy, by reducing the SCC from 600 000 to 300 000 for a herd of 100 dairy cows at 34.0 €/100 kg of milk (purchase price of milk in Slovakia), the farmer can obtain up to 6 290 €/year by reducing the loss of milk production in the same herd conditions and feed [(52), Table 1].

#### Nutrition

Having a healthy herd with proper nutrition is the first step to becoming a successful breeder. A balanced diet plays an important role in udder resistance to infection because certain nutrients affect various mammary resistance mechanisms, namely: (1) leukocyte function, (2) antibody transport and (3) mammary tissue integrity (57, 58).

Cattle breeders with correctly compiled and mixed feed ration, which meets all the requirements imposed during the individual stages of lactation (phase nutrition) can achieve increased resistance of dairy cows to mastitis-causing pathogens. When composing and mixing the feed ration, it is necessary to use feeds that are safe from fungi and mycotoxins. Contaminated feeds adversely affect the immune system, weakening it, hence, making it unable to defend itself against pathogens entering the udder. Similarly, a similar effect on immunity is caused by an overall impoverished feed ration for energy, nitrogenous substances and other essential nutrients necessary for the proper functioning of the body.

Not only can dietary nutrients have a direct impact on immune function and susceptibility to mastitis, but they can indirectly increase cow susceptibility to mastitis through their impact on peripartum metabolic diseases. All essential nutrients can induce one or more metabolic diseases when either deficient or in excess in the transition diet. Hypocalcemia (milk fever) has been shown to slow the closure of the teat sphincter. Cows with milk fever are 8.1 times more likely to have mastitis and nine times more likely to have a coliform mastitis event as a result. Mastitis was also associated with ketosis and retained placenta. Cows with fatty infiltration of the liver have been shown to be slower in clearing E. coli from their mammary gland. Consequently, this translates that a feed ration with the necessary amounts of vitamins, minerals and other immunostimulatory additives improves the body's defenses against pathogenic bacteria (59, 60).

Recently, when compiling feed rations, breeders use various feed additives in the form of humic acids (uptake of mycotoxins, optimization of rumen pH, stabilization of symbiotic microflora and increased utilization of nutrients) (61, 62) or mineral-vitamin supplements (vitamins E, C, and A and essential trace elements; zinc - Zn, copper - Cu, and selenium - Se) with significant antioxidant and immunostimulatory effects to reduce the risk of mastitis in the postpartum period (63–66).

##### Vitamin E and Selenium

Vitamin E and selenium (Se) compounds are among the most effective antioxidant nutrients, although often deficient in compound feed during the dry period and peripartum period. Selenium is a vital component of the antioxidant enzyme glutathione peroxidase, which is essential for the protection of cells and body tissues. The beneficial effects of Se can be attributed to the decreased damage to cells by oxygen radicals and peroxidases with an increased efficiency of the enzymes that are involved in intracellular killing mechanisms (67).

The proportion of Se in grains or in forage depends on the presence of this element in the soil. The Se concentration in the soil varies greatly even over relatively small areas. Because of this, Se supplement is recommended to maintain the minimum consumption level and to ensure effective immune response. According to the National Research Council (68), the Se recommendations for dairy cattle vary from 0.1 to 0.3 mg/kg DM, regardless of the age and the physiological state of the animal, but there is no reference to the form in which Se supplement should be used, i.e., organic or inorganic. The most used Se inorganic forms are sodium selenite and sodium selenate. In feeds in general, and in yeasts, Se is found in the organic form (69).

Erskine (57) and Hogan et al. (70) documented that Se deficiencies in compound feed can have serious consequences on MG health. The same authors confirmed the benefits of dietary supplementation with organic as well as anorganic Se sources for the control of bovine mastitis. Neutrophil killing of *S. aureus* and other environmental pathogens (e.g., *E. coli*) is greatly enhanced for dairy cows receiving an Se supplement compared with cows that were deficient in Se. Erskine (57) also showed that the supplemented cows experienced clinical mastitis of lesser severity and shorter duration than those of unsupplemented cows. Similarly, Sordillo et al. (71) reported a decrease of the phagocytic ability of blood and milk neutrophil to kill pathogens in dairy cows with a Se deficiency. The opposite situation was reported for neutrophils cows having a higher status of selenium.

According to Slavik et al. (65), Se-organic dietary sources (Se enriched yeast) are more effective than sodium selenite for increasing the concentration of Se in blood, colostrum and milk. On the other hand, other studies confirm that there are no differences between the supplementation of organic and inorganic forms of selenium. Oltramari et al. (72) in his study demonstrated that dairy cows supplemented with organic Se and in organic Se during 124 days showed a reduced incidence of subclinical positive mastitis and strongly positive mastitis as decreased SCC compared to control group feed with 0.278 mg.kg^−1^ DM of Se during experimental phase. Similar results as in the previous study were verified in study by Viero et al. (73) using doses of sodium selenite and selenium yeast.

From our previous studies suggest that the supplementation of diets for dairy cows, is not important form of Se but its length of supplementation. Ideally, when elevated concentrations of Se (0.3–0.5 mg.kg^−1^ of DM) are added to the feed throughout the dry period and first stage of lactation. Due to the increased immunostimulatory effect of Se is good to add vitamin E to the feed ration (67, 69).

Vitamin E, which is similar to Se in its biological properties. Is an important component of all cell membranes and provides stability and prevents the debilitating peroxidation of membrane lipids. Vitamin E also plays a regulatory role in the biosynthesis of various inflammatory mediators that are necessary for the integrity of integument and wound healing and has shown increased neutrophil bactericidal activity (71).

This essential antioxidant is found in high quantities in fresh, green food stuffs; however, the concentration of vitamin E decreases as age of plants and length of storage increase, and vitamin E is often destroyed in silages. Therefore, deficiencies are common in unpastured cows and in cows during seasons when pasture is not available (74).

Research, as well as practical results, show that Se in particular, together with vitamin E, have a synergistic effect, reducing the prevalence of clinical mastitis, new IMI at calving, and SCC, as well as reducing the severity and duration of clinical mastitis to a greater degree than the supplementation of either micronutrient alone (69, 70, 75).

Selenium and vitamin E are part of the protection of individual antioxidant levels of cellular structures against the amount of free radicals formed, maintaining low tissue concentrations of reactive oxygen species, which are beneficial for the body in many cases. Moreover, they are also used in the etiopathogenesis of diseases and pathological processes (74, 76).

Deficient intake of Se and vitamin E in feed rations and a long-term decrease in plasma concentrations (Table 2) of these two important nutrients, which are part of the body's antioxidant system, lead to increased lipid peroxidation and damage to cell membranes (67).

**Table 2:** Overview of Se and vitamin E deficiency syndromes in ruminants.

**Species**	**Syndrome**	**Affected system, resp. organ**
Cattle	Nutritional myodystrophy of calves	Skeletal muscle, myocardium
	Retained placenta	Placental connection with the uterus
	Ovarian cysts	Ovaries
	Decreased production, mastitis	Udder, mammary gland
	Immune system disorders	Decrease Th lymphocyte production and phagocytic activity
	Anemia	Erythrocytes
Sheep/goat	Nutritional myodystrophy	Skeletal muscle, myocardium
	Infertility	Loss of uterine tone
	Decreased production, mastitis	Udder, mammary gland
	Immune system disorders	Decrease Th lymphocyte production and phagocytic activity

*Source: Adapted from Zigo et al. (67)*.

According to Hogan et al. (70) vitamin E and Se deficiency in cows leads to increased formation and accumulation of peroxide radicals in tissues and lipid structures, resulting in placental retention, MG swelling and an increased incidence of mastitis. In dairy cows with a low intake of vitamin E at a dose of 20 IU/kg of dry matter (DM) and 0.1 mg Se/kg of DM during the dry period, incidence of mastitis was increased by 57% compared to the group of cows fed 50 IU/kg of DM vitamin E and 0.3 mg Se/kg of DM.

Eulogio et al. (77) demonstrated that the incorporation of Se and vitamin E in commercial diets of grazing first lactation cows increases milk production and percentage of crude protein, solids non-fat and lactose content and decreases SCC. This data confirms earlier findings that Se and vitamin E supplementation are related to mammary health gland. The performance and economic feasibility of the use of Se plus vitamin E allowed us to obtain a profit margin of $ 0.21 per animal per day in this study.

It seems that Se and vitamin E should not only be supplemented and determined in the feed ration but also monitored in the blood plasma of animals. Breeders often rely on the feed intake, but even increased supplementation of vitamin E and Se in the form of concentrates and premixes may not correlate with their current blood concentrations, as was demonstrated in our previous study in cows during the dry and postpartal period. Significant decrease in plasma concentrations of Se and vitamin E below the recommended physiological range during the dry period cannot be compensated by an increased supplementation in feed ration. Rapid rise in plasma concentrations of Se and vitamin E is best ensured by parenteral administration, while long-term stabilization may be achieved by feeding ratios with an increased content of these antioxidants throughout the dry period (67, 69).

In dairy cows, a minimum daily intake of vitamin E from a feed ration of 500–600 IU/head and Se of 0.1–0.3 mg/kg DM is recommended to maintain optimal health. In dry cows and at the initial phase of lactation, daily vitamin E supplementation of 1,000–2,000 IU/head should be provided and the feed ration should contain 0.3–0.4 mg Se/kg DM to achieve a positive effect on the health of the MG and reproduction (69).

##### Humic Acids

In addition to the supplementation of mineral and vitamin supplements, humic acids have been added to feed ration in recent years to increase the body's defenses and eliminate adverse conditions that could lead to the occurrence of various diseases and ailments. Humic acids are natural organic substances that are formed by the chemical and biological decomposition of organic matter of plant origin and synthetic activity of microorganisms. It alongside fulvic acids and humin are among humic substances that are part of humus. They are based on lignin collectively with other components of plant biomass (sugars, fats, proteins, waxes and resins) (78).

The increased use of humic acids in animal nutrition is further exacerbated by the fact that from 28th January 2022, the legislation will be applied in all Member States of the European Community prohibiting the preventive and mass administration of antibiotics for all groups of farmed animals. In practice, this means continued administration of antibiotics to sick animals, however, only individually, with a clinical examination performed before their administration, respecting the withdrawal period for animal products after their administration. Oral administration of humic acids is one of the approved real alternatives to antimicrobials and zinc oxide (79).

Due to increasing milk production to its maximum level and the associated risk of intramammary infections with subsequent antibiotic treatment, the addition of humic acids to the feed ration is increasingly used, especially during the drying period and the first half of lactation. Rich nutrition with nuclear feeds (especially in the first 100 days of lactation) with a high content of protein, energy and at the same time, low fiber content, which in the case of ruminants is unnatural, negatively affects their health, reproductive and economic indicators (80). Addition of humic acids in a daily dose of 100 g per dairy cow for 60–70 days leads to decreased SCC, reduction in the incidence of subclinical mastitis as well as an increase in both protein levels and fat by 0.2–0.5% and 0.3–0.5%, respectively (81).

Furthermore, the supplementation of humates to the feed stimulates the immune system and the growth of symbiotic rumen microflora. Their mechanism of stimulation of the immune system is related to the ability of humates to bind sugars in the body to complexes. A large number of these complexes allow the body to synthesize glycoproteins that bind to NK and T cells as a modulator and communication link between cells. Thus, they regulate the immune system and prevent the imbalance of T and NK cells (61).

Humates added to feed ration stimulate the growth of symbiotic microorganisms depending on the species while suppressing pathogenic microorganisms. Species that have been inhibited by natural humic agents include *Candida albicans, Enterobacter cloacae, Proteus vulgaris, Pseudomonas aeruginosa, Salmonella typhimurium, Staphylococcus aureus, Streptococcus epidermidis*, and *Streptococcus pyogenes* (62).

#### Herd Environment and Management

**Figure 5:**
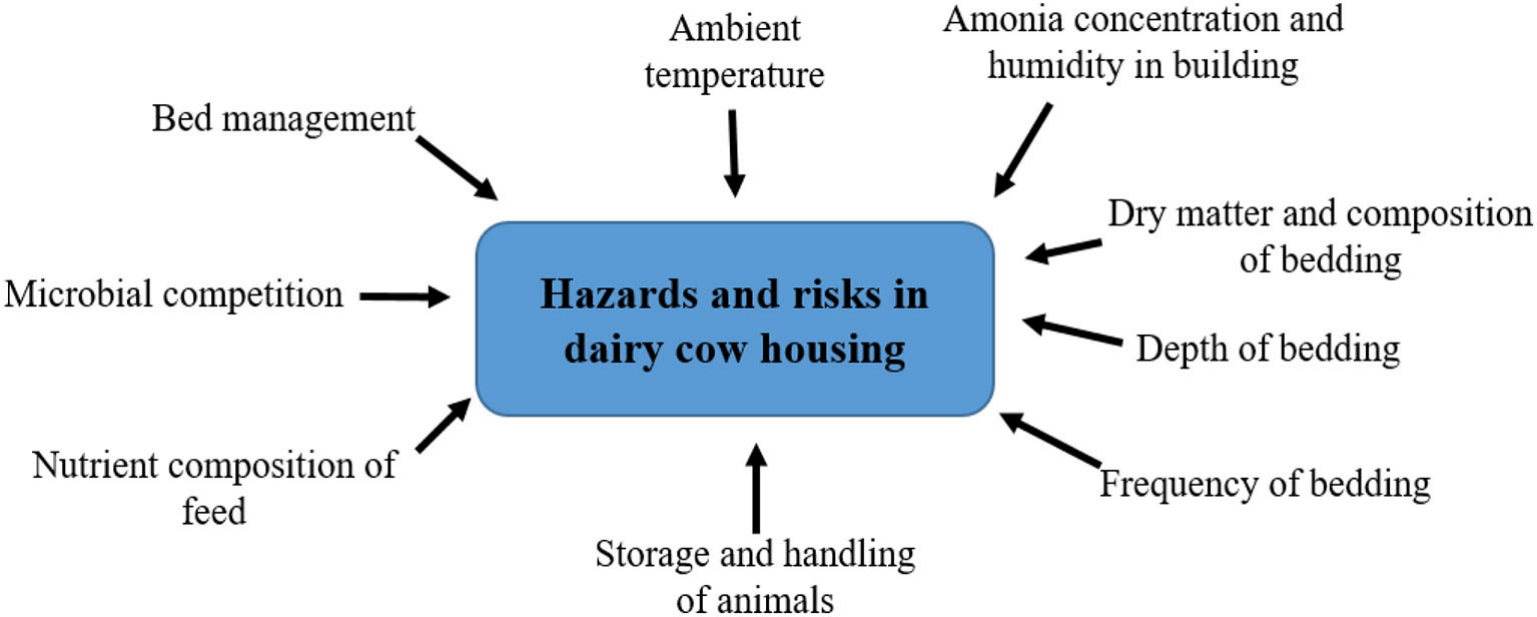
Factors affecting hazards and risks associated with bedding materials in dairy cow housing. Source: Adapted from Bradley et al. (82).

The cleanliness of the environment in which the animals are located is important for the improvement of the health of the udder and elimination of mastitis. The main influences on the hazards and risks associated with aspects of housing and its management are illustrated in [(82), Figure 5]. The aim of daily care and maintenance of the stalls is to have clean, dry and satisfied dairy cows when entering the milking parlor. The occurrence of environmental mastitis (environmental mastitis) is related to the level of housing hygiene. From this viewpoint, achieving the lowest possible pollution of the body, especially the udder, deserves high priority in the breeding of high-yielding dairy cows (83).

Among the main actions that help to reduce environmental pathogens is the regular replacement of bedding (straw, sawdust) and the removal of manure. The bed must always be dry and clean to prevent the formation of a breeding ground for bacteria (fecal enterococci and streptococci), which causes environmental mastitis. It is good to use lime or special commercial products available on the market to disinfect and absorb excess moisture from the lying boxes (22, 84).

Due to the frequent lack of straw, recycled manure solids (RMS) have been used as a substitute bedding material in recent years to create sufficient comfort for dairy cows. RMS consists of dry matter and a nutrient-rich fraction obtained by mechanical or gravitational separation of slurry manure removed from dairy cow housing systems. To ensure its hygienic quality and optimum pH, RMS is often combined with straw and other components such as limestone or zeolite (85, 86).

In our previous study, improved composition of bedding per one cubicle consisted of ground limestone (100 kg), water (80 L), recycled manure solids (15 kg), and straw (25 kg) influenced the level of hygiene on indicator microorganisms in comparison to conventional straw bedding. Samples of lying boxes with improved bedding showed reduced total viable count, coliform bacteria, fecal coliform bacteria and fecal streptococci in one-day-fresh improved composition bedding as well as the first 2 months after it was laid. In addition to reducing the number of microorganisms, by using the improved composition bedding for a period of 3 months, the effect of reduced infection pressure from the environment was demonstrated, which resulted in an increased number of healthy quarters with negative CMT score and a reduced incidence of subclinical mastitis in dairy cows (87).

More so, regular cleaning and disinfection of the milking parlor and waiting room should be observed. Usually, the cleanliness of the housing of heifers and dried cows is neglected, making room for pathogens to enter the MG. In herd management, it is important to separate mastitis or otherwise sick dairy cows from healthy animals until they are completely cured or eliminated. Additionally, it is likewise ideal to create a group for primipary cows to prevent the transmission of infection from cows (22).

#### Lactating Cows and Milking

During this period, the dairy cows are inspected based on a once a month performance check, giving us a detailed picture of SCC, while highlighting the level of California mastitis test (CMT) needed on the farm (88). At the end of the colostrum period on the 4–6th day, it is necessary to examine each dairy cow by CMT, that is, upon which only healthy animals are moved to the production group. In case of a positive CMT result, it is necessary to proceed with a possible treatment according to Figure 4. An important outcome is also the early culture of positive samples from cows with subclinical mastitis based on the positive CMT (89).

This form of mastitis is the most prevalent type of intramammary infection, but it cannot be detected by looking at the MG or the milk because both appear normal. The majority of infections are caused by the staphylococci and streptococci. If antibiotic therapy is to make a significant contribution toward reducing the herd level of mastitis as well as the bulk tank SCC, it is necessary to treat subclinical infections as well as the clinical cases. It is not unusual to have 15–40 subclinical cases for every clinical case caused by contagious pathogens. Generally, antibiotic intramammary therapy of subclinical mastitis during lactation is indicated only when *Strep. agalactiae* or *S. aureus* are present, or the producer is in danger of losing his milk market due to a high bulk tank SCC (58).

Blowey and Edmondson (1) observed that the treatment of cows subclinically infected with *Strep. agalactiae* is usually successful and results in increased production and a dramatic decrease in bulk tank SCC. In contrast, it is not considered cost-effective to treat cows that are chronically infected with *S. aureus* because cure rates during lactation are rather poor.

A proper milking hygiene program that meets all biological and hygienic requirements of the dairy cow significantly influences the maintenance of good udder health. The purpose of milk hygiene is, of course, not just the control of mastitis in the cows, but to guarantee that the milk sent for sale is fit for human consumption. This requires attention to hygiene at all stages of the milking process and storage in bulk tanks. The standard measure of the milk hygiene is the total bacteria count (TBC). This is measured routinely by the dairies collecting milk off farm. Within the EU, herd counts of TBC >100 000 per ml are penalized to the extent that action is called for; TBC >50 000 may incur a reduction in the milk price. A marker of good practice would be TBC that were consistently <15 000/mL (20).

**Figure 6:**
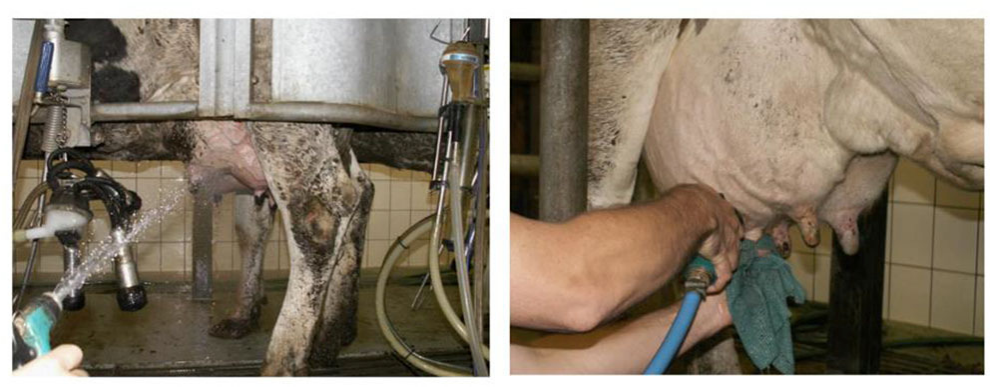
Incorrect and correct udder washing. Note: Direct spraying with a strong stream of water on the udder is unacceptable. Slightly flowing hot water is possible to use only on contaminated teats of the udder. Source: Tančin (64).

The most common bad practices and mistakes in milking hygiene program are: spraying water on the udder when the cows enter the parlor (Figure 6), emptying the teat cistern (first sprays of milk) on the ground, weak stimulation and insufficient udder toilet (ineffective predipping) before milking, soiled and unwashed milking clusters of the milking machine, attachment of the milking cups to a dirty or insufficiently wiped udder, incorrect attachment of milking machines, failure to disinfect the teats after each milking, too short or ineffective postdipping, etc. (48).

Milkers must follow a well-defined workflow that includes conditions associated with milking on a particular business; these are divided into the following steps: (1). washing and drying of teats, (2). make the first sprays from all the quarters in a container with a black bottom and perform a sensory assessment of the quality of the milk, (3). application of pre-dip (preparation before milking), (4). drying of teats, (5). put on the milking equipment, (6). no milking on dry, (7). post-dip application (preparation after milking), (8). rinsing and regular technical maintenance of milking equipment, (9). after milking, feed the cows to keep them upright until the teat close (~20–30 min.) (40).

Other important factors in milk production are milking efficiency and regular maintenance of the milking equipment. Mistakes and undesired effects of machine milking that increase the risk of colonization of the teat duct and new IMI include: incomplete milking of the udder, incomplete empty milking, large pressure fluctuations in vacuum regulator, too fast or too slow pulsation, slow flow of milk from the milk claw distributor causing “flooding of teats,” removing the milking clusters before switching off the vacuum, etc. It is also necessary to keep in mind the service life of the individual components of the milking equipment as well as the service and setting the functional parameters of the milking equipment. Any underestimation or delay in regular inspections of the milking equipment to “save money” later draws much more money out of the cash register (48).

#### Drying Cows

A very important part of the further milk production after the previous lactation is the management in the dry period. This is a period when ideal conditions are created for the regeneration of MG tissue after previous production, on many aspects–physiological, morphological and immunological (90, 91).

Cows are naturally protected against intramammary infections during the dry period by formation of a keratin plug in the teat canal. However, time of teat canal closure varies among cows. In a study conducted by Williamson et al. (92), 50% of teat canals were classified as closed by 7 days after dry off, 45% closed over the following 50–60 days of the dry period, and 5% had not closed by 90 days after dry off. Teats which do not form a plug-like keratin seal are thought to be most susceptible to infection.

However, a quarter that becomes infected during the dry period, or that remains infected from the previous lactation, will produce 30–40% less milk (54). One of the most important aspects in this period is the length of the drying interval of the cows. Cows should be dried no later than 60 days before planned calving, except for dairy cows with a high daily intake (more than 25 L), where the dry period can be reduced by 10 days. The risk of mastitis is greatest at the beginning and end of this period; this is the reason, it is necessary to pay extreme attention to pregnant animals with proper hygiene procedures and principles of application of intramammary preparations (93).

Dairy cows ready for drying must be examined by CMT, if they are positive, they must be treated and only then dried with an effective intramammary long-term effect antibiotic. Treatment of all quarters of all cows at drying off (blanket dry cow therapy) is one of the most important components of a comprehensive plan of mastitis control. This is because dry cow therapy both cures existing infections, caused mainly by contagious pathogens, and prevents the development of new infections, caused mainly by environmental pathogens (5, 94).

For drying cows, a “Combo” application therapy for the administration of preparations based on antibiotics and keratin seal is effective. After the last udder milking, it is necessary to administer intramammary antibiotic injections for drying and injections with teat sealant providing an external physical barrier for the teat orifice during critical times in the dry period. Thereafter, it is recommended to soak the teats in a post-dip. When a cow is prepared in this way, the onset of a new infection during the dry period would be prevented (27).

**Figure 7:**
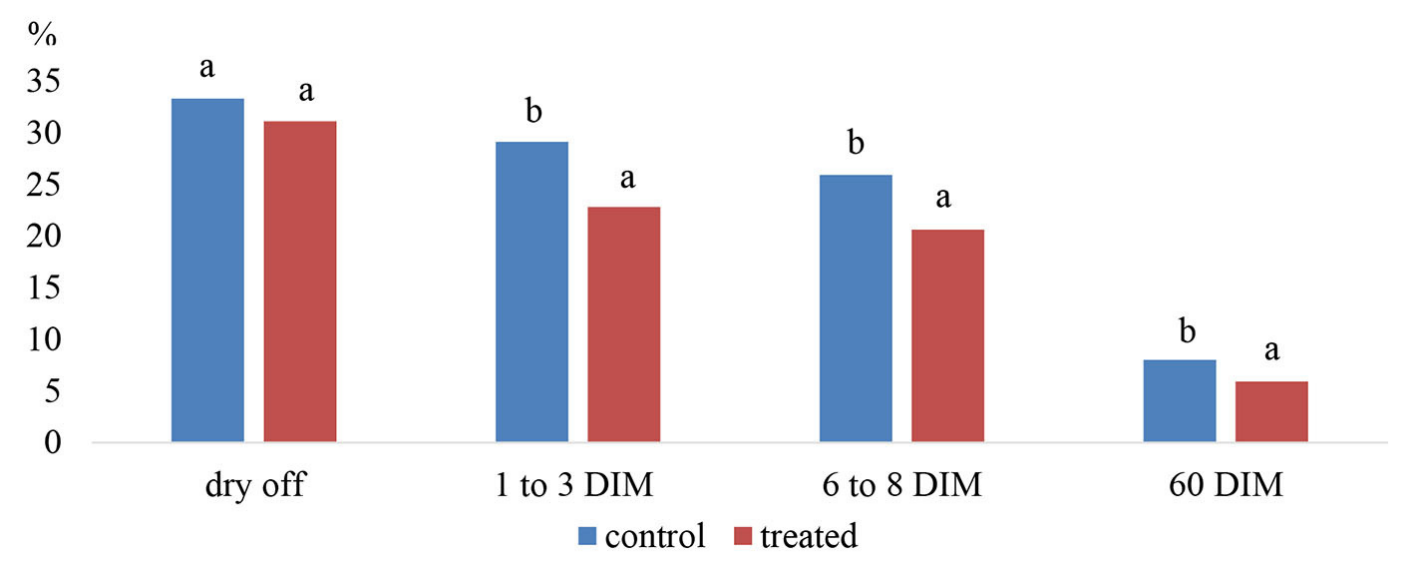
Prevalence of IMI in control (antibiotic only) and treated (seal plus antibiotic) quarters at dry off, 1–3 days in milking (DIM), 6–8 DIM and 60 DIM. Note: ^a,b^% of all quarters with IMI between columns are significantly different (*P* < 0.05). Source: Adapted from Godden et al. (95).

Godden et al. (95) demonstrated a significant reduction in the incidence of clinical mastitis during the first stage of lactation in cows that had been treated with intramammary anantibiotic with teat seal when compared to cows treated only teat seal. Their results confirmed that intramammary infusion with teat sealant as an adjunct to long-term effect antibiotic at dry off had a significant effect on reducing the risk for acquiring a new IMI between 1 and 3 days in milking (DIM; treatment = 22.8%, control = 29.1%), 6–8 DIM (treatment = 20.6%, control = 25.9%) and 60 DIM (treatment = 5.9%, control = 8.0%) (Figure 7).

Legislative and consumer pressure is to reduce antibiotic use in primary milk production. One way to achieve that is to selectively use antibiotics in dry cows. Selective use of antibiotics can be a useful tool, but only in individual quarters of the udder or in whole udders with low SCC and without the presence of pathogens. In farming conditions selective application of antibiotics to dry cows is done on the basis of a history, clinical examination of the udder and evaluation of the SCC from milk utility monitoring records (96).

After considering all aspects, breeders classify dairy cows into three categories. The first category includes dairy cows in which no problems with MG health were observed during lactation, the SCC in the sample taken before drying did not exceed 200 x 10^3^ in 1 mL and no clinical or subclinical form of mastitis was currently diagnosed. These dairy cows are ideal for drying without the application of antibiotics using a teat sealant (it definitely closes the teat canal and prevents pathogens from the entering MG) or by immersing the teats in a protective solution. The second group consists of dairy cows that have an SCC higher than 200 x 10^3^ in 1 mL, in which mastitis was recorded during lactation, but currently the MG is free of clinical signs of inflammation. In this case, a long-acting intramammary antibiotic is applied to each quarter in conjunction with an internal teat sealant or a protective solution. The antibiotic for dry period should be chosen based on the overall susceptibility of the bacteria to the antibiotics used (antibiogram) on the farm as well as on the last examination of milk samples from individual dairy cows (56, 93).

The third group includes dairy cows with current clinical or chronic mastitis as well as dairy cows with non-milked quarters to which an antibiotic preparation may be applied. In these dairy cows, the infected quarters must first be treated during lactation with adequate intramammary antibiotics and proper milking. Subsequently, the cow is dried according to the above scheme for the second group with an intramammary antibiotic preparation and teat sealant (97, 98).

Intramammary treatment with blanket drying-off therapy of all udder quarters with long acting antibiotics at the beginning of the dry period, has been recommended for a long time because it proved to reduce efficiently the level of infection at the herd level. The products used are mainly effective against Gram positive bacteria, yet there is no increase in the prevalence of infections by Gram negative bacteria associated to their use. After the cessation of treatment and before calving, the cow is at risk of developing a new infection when it is no longer under antibiotic protection. The antibiotic concentrations more than 3 weeks during drying period has the potential to interfere durably with any protective intramammary microbiota, and microbiota disruption by antibacterial products is known to favor dysbiosis, which may increase the susceptibility to infections. At this point, sterile udder and created colostrum that are an ideal breeding ground for bacteria, is beginning to form (42).

For these purposes, probiotics have been used to protect the udder and support the protective microflora. According to the concept of mastitis as a manifestation of dysbiosis, i.e., an imbalance of the intramammary microbiota, the use of probiotics to re-equilibrate the microbiota appears as a possible corrective measure. Oral supplementation of probiotics for the treatment of IMI have not been effectively in polygastric animals such as ruminants, especially since the enteromammary pathway is poorly operative in these species. This is probably why probiotics for the bovine MG have been administered through the teat canal (99).

Several Lactobacillus species as *L. lactis, L. acidophilus, L. casei* or *L. perolens* has been used as an alternative non-antibiotic treatment of mastitis. Intramammary inoculation of a probiotic mixture to cure mastitis has been found to be efficient only in minor pathogens as *Corynebacterium bovis* and coagulase-negative staphylococci. Intrammary infusion of 10^6^ cfu *L. perolens* or other probiotic mixture has been found ineffective to major pathogens such as *S. aureus*, streptococci and *E. coli*. Application of probiotics through teat canal causes an inflammatory response from the MG, and this is probably why they have been used for therapy rather than for prevention of IMI. Although quite a few studies reported some protective effect by minor pathogens, others have found the converse or no effect, and several recent reviews conclude that a protective effect would be of low magnitude in any case (100, 101).

#### Vaccination Procedures Against Mastitis Pathogens

Immunoprophylaxis of mastitis involves means and methods for targeted enhancement of specific immunity to an infectious agent. The possibility of vaccinating cows against specific pathogens causing intramammary infections is a relatively new specific tool for suppressing, controlling and preventing MG inflammation, but this method also has its drawbacks (102).

Most vaccines are based on achieving high levels of specific antibodies in the blood of dairy cows that pass into milk. The first drawback is that the transfer of antibodies from the blood to the milk does not take place by diffusion. It occurs by active transport independent of the concentration of antibodies in the blood and is dependent on the physiological state of the MG (103).

Another drawback is the low level of complement in the milk and the low performance of neutrophils. Their lack of performance is due to the fact that they only express a small percentage of immunoglobulin receptors on their surface. The fact that they consume a high amount of oxygen limits their function due to the fact that the oxygen concentration in milk is a 100 times lower than in blood. In addition, phagocytic cells need energy for the process of phagocytosis, which they can draw from glucose, but this is also present in low concentrations in milk. Another problem is that a large part of phagocytic cells also absorbs harmless fat droplets, which depletes their number (104).

Monovalent (*Staphylococcus aureus*) or polyvalent (streptococci/staphylococci) vaccines can be used in a mastitis control programs. Available vaccines can shorten the duration of infection and limit the circulation of some contagious microorganisms causing MG inflammation, mainly *Staphylococcus aureus*, throughout the herd. In the USA for example, four preparations are used which, although do not reduce the frequency of MG infections, they alleviate its manifestations (105).

Toušová et al. (106) recommends the use of a polyvalent vaccine in dairy herds with recurrent mastitis and to reduce clinical signs caused by coliform bacteria, *S. aureus* and coagulase negative staphylococci. The author also reports a significant reduction in SCC in a pooled milk sample during lactation in vaccinated dairy cows throughout lactation. At the same time, it was found that dairy cows treated according to the vaccination schedule in three doses showed a reduction in the incidence of mastitis up to 43.5%, in contrast to untreated dairy cows, when the incidence was 65%.

According to Doležal et al. (107) most vaccination schedules consist of three doses. The first dose is given when the dairy cows are dried, the second a month later and the third 2 weeks after birth. The efficiency of immunoprophylaxis in dairy cows at second and higher lactations is reported to be in the range of 10 to 20%, both by reducing clinical forms of mastitis and also by reducing the number of subclinical and latent mastitis. In order to achieve the maximum effectiveness of immunoprophylaxis, the author recommends starting vaccination in heifers before the first mating. The first dose is given at 6 months of age and the next one in 14 days. This procedure is repeated every 6 months until calving. Then, revaccinations occur at calving and 6 months after calving. The author further states in his study that after five administered doses, the primipary cows in the calving period has a significant reduction in the incidence of mastitis.

Although preventive use of the vaccine in production herds is an economic burden associated with higher costs of purchase and self-administration, its positive benefits associated with better milk monetization due to reduced SCC with lower mastitis is one way to improve profitability of milk production, healthy cows and reduces the number of weaned dairy cows (105).

## Conclusion

Taking into account all measures affecting the reduction of mastitis, effective prevention programs can be developed. One of the most proven antimastitis programs is based on strict adherence to ten steps, which include: (1). setting a mammary gland health target, (2). ensuring clean and dry housing of animals, (3). adherence to the order of dairy cows in the milking parlor (calved cows, production groups, end of lactation and treated), (4). correctly chosen milking procedure, (5). care of milking equipment, (6). initiation of early and adequate treatment of clinical cases of mastitis, (7). keeping records of treated cows with evaluation and updating of antibiotics used, (8). effective management and selective use of antibiotics in the drying off cows, (9). culling of the chronically ill resp. incurable dairy cows, (10). periodic assessment of the antimastitis program.

These 10 measures in the antimastitis program provide a comprehensive system that allows breeders to control the most important actions that are directly related to the origin and spread of mastitis in the herd. Only respect for current scientific knowledge in a logical context and the complex in the daily application of proven prevention and control practices in production farms can positively affect the overall production, quality and nutritional value of milk with a positive impact on consumer health.

## Author Contributions

FZ proposed and wrote the manuscript. JB and EP-K provided critical review the manuscript. MV provided helpful comments on the article. SO editing the manuscript. JV and FZ corrected manuscript according to the reviewers' recommendations. All authors contributed to the article and approved the submitted version.

## Conflict of Interest

The authors declare that the research was conducted in the absence of any commercial or financial relationships that could be construed as a potential conflict of interest.
